# Chemical Profile and In Vitro Gut Microbiota Modulation of Wild Edible Mushroom *Phallus atrovolvatus* Fruiting Body at Different Maturity Stages

**DOI:** 10.3390/nu16152553

**Published:** 2024-08-03

**Authors:** Raweephorn Kaewsaen, Santad Wichienchot, Parinda Thayanukul, Suvimol Charoensiddhi, Wasaporn Preteseille Chanput

**Affiliations:** 1 Department of Food Science and Technology, Faculty of Agro-Industry, Kasetsart University, 50 Ngam Wong Wan Rd., Ladyao, Chatuchak, Bangkok 10900, Thailand; raweephorn.k@gmail.com; 2 Center of Excellence in Functional Foods and Gastronomy, Faculty of Agro-Industry, Prince of Songkla University, Hat Yai, Songkhla 90110, Thailand; santad.w@psu.ac.th; 3 Department of Biology, Faculty of Science, Mahidol University, Bangkok 10400, Thailand; parinda.tha@mahidol.ac.th; 4 Center of Excellence for Vectors and Vector-Borne Diseases, Faculty of Science, Mahidol University, Salaya, Nakhon Pathom 73170, Thailand

**Keywords:** bamboo mushroom, beta-glucan, gut microbiome, maturity stage, short-chain fatty acids

## Abstract

*Phallus atrovolvatus*, a wild edible mushroom, has attracted increasing interest for consumption due to its unique taste and beneficial health benefits. This study determined the chemical components in the so-called fruiting body during the egg and mature stages and investigated its gut microbiota-modulating activities. The egg stage contained higher total carbohydrates, dietary fiber, glucans, ash, and fat, while the total protein content was lower than in the mature stage. Two consumption forms, including cooked mushrooms and a mushroom aqueous extract from both stages, were used in this study. An in vitro gut fermentation was performed for 24 h to assess gut microbiota regulation. All mushroom-supplemented fermentations increased short-chain fatty acid (SCFA) production compared to the blank control. Furthermore, all mushroom supplementations promoted the growth of *Bifidobacterium* and *Streptococcus*. Samples from the mature stage increased the relative abundance of *Clostridium sensu stricto 1*, while those from the egg stage increased the *Bacteroides* group. The inhibition of harmful bacteria, including *Escherichia-Shigella*, *Klebsiella*, and *Veillonella*, was only observed for the mature body. Our findings demonstrate that *P. atrovolvatus* exhibits potential benefits on gut health by promoting SCFA production and the growth of beneficial bacteria, with the mature stage demonstrating superior effects compared to the egg stage.

## 1. Introduction

A mushroom is the fruiting body of a fungus, typically grown in soil or its food materials [1]. Mushrooms have been widely consumed as a food item for centuries due to their nutritional value, delicious taste, and health-promoting benefits. The global production of cultivated edible and medical mushrooms has increased more than 30-fold since 1978 [2]. Thus, studies on the health benefits of mushrooms have been gaining interest.

The mushroom cell wall contains β-glucan, a polysaccharide consisting of D-glucose monomers linked by β-1,3 glycosidic bonds as the backbone, with β-1,6 branch linkages [3]. β-glucan is not digested in the human gastrointestinal tract because the digestive enzymes secreted by the pancreas and brush-border epithelial cells cannot hydrolyze β-glycosidic bonds. Hence, β-glucan is resistant to digestion in the upper gastrointestinal tract and remains in the large intestine, where it can be utilized by the gut microbiota [4]. 

The gut microbiota comprise a complex community of 100 trillion microbes, with more than 1000 microbial species colonizing the human intestines. Most gut microbiota are obligate anaerobes, including Firmicutes (65%), Bacteroidetes (25%), Proteobacteria (8%), and Actinobacteria (5%) [5,6]. These gut microbes can ferment non-digestible polysaccharides because they have multiple metabolic genes encoded by the genome and produce several beneficial metabolites for the host [7]. The gut microbiota and their metabolites play important roles in various health benefits, including nutrient metabolism, glucose homeostasis, lipid metabolism, inhibition of pathogens, gut barrier integrity, regulation of immune responses, balance in mood disorders, and alleviation of neuroinflammation [8,9]. One class of metabolites produced by the gut microbiota are the SCFAs, which are volatile fatty acids, among which acetic acid, propionic acid, and butyric acid are the most abundant. Several studies have demonstrated that SCFAs are one of the most important microbe metabolites and are especially related to gut health. For instance, acetic acid plays an important role in inhibiting pathogenic bacteria. Propionic acid is involved in gut hormone stimulation. Furthermore, butyric acid increases mucin production, enhances epithelial barrier function, and is the main energy source of colonocytes [9,10].

The composition and abundance of colonic bacteria are affected by several factors, including genetics, the mode of delivery, medication, lifestyle, and diet patterns [11]. Therefore, one’s diet, especially dietary fiber, impacts the gut microbial community and gut homeostasis. β-glucan from mushrooms is one type of dietary fiber and is well-known for its prebiotic potential that shapes the gut microbial composition and contributes to health benefits, particularly in gut health [12]. Overall, previous findings have revealed that mushroom β-glucan-supplementation in a colitis mouse group increased the abundance of beneficial bacteria and inhibited the growth of pathogenic bacterial groups. Taken together, the production of SCFAs, including acetic acid, propionic acid, and butyric acid, was promoted, resulting in a reduction in intestinal inflammation-related disorders [13,14,15]. These studies have demonstrated that β-glucan from typically consumed mushrooms could alleviate gut inflammation via the modulation of intestinal microbes. Thus, further explorations of new edible mushrooms and their stages could expand knowledge about their positive effects on gastrointestinal health.

*Phallus atrovolvatus* (Syn. *Dictyophora duplicata* (Bosc.) E. Fisch.) is found in the southern forests of Thailand and has been recently identified as a new Thai strain. This mushroom has been histologically consumed by locals for decades without reports on any toxicity or discomfort after consumption. Although *Phallus* spp. are common edible wild mushrooms and regularly used in many cuisines [16,17], it is worth noting that not all wild mushrooms are edible; some of them can cause life-threatening issues. Nowadays, *P. atrovolvatus* can be commercially cultivated through the support of the Department of Agriculture, the Ministry of Agriculture and Cooperatives, Thailand. *P. atrovolvatus* is scientifically close to *P. indusiatus*, mostly found and widely consumed in China. These two mushrooms are commonly called ‘bamboo mushroom’. The Chinese strain of the bamboo mushroom *P. indusiatus* is regarded as the queen of mushrooms due to its nutritional value, taste, and unique appearance, and it also exhibits several health-promoting benefits, including antioxidative, anti-cancer, anti-inflammatory, neuroprotective, immunomodulatory, and gut microbiota-modulating effects [18].

The biological properties of the Thai strain of bamboo mushroom, *P. atrovolvatus*, have recently been reported. Crude polysaccharide, extracted from the fruiting bodies of *P. atrovolvatus* using hot water extraction, showed strong antioxidative activities in vitro and alleviated gut inflammation via the inhibition of myeloperoxidase activity and pro-inflammatory cytokines in dinitrobenzene sulfonic acid-induced colitis mice [19,20]. In Thailand, *P. atrovolvatus* is commonly consumed as the mature fruiting body; however, consumption at the egg stage has attracted interest due to its unique and specific texture. The growth of the bamboo mushroom’s fruiting body is divided into four maturity stages, starting from primordia and developing into ball-shaped, peach-shaped, and mature stages [21]. The immature stage, which is peach-shaped, is also called the egg stage. Different maturity stages of mushrooms can affect their taste, flavor, chemical compounds, polysaccharide contents, and biological properties [22]. A previous study reported that the total phenolic and flavonoid contents of *Ganoderma lucidum* stipes were highest in the spore maturity stage. In contrast, the fruiting body maturity stage contained the highest content of ganoderic acid, one of the triterpenoids found in *Ganoderma* spp. mushrooms [23]. The chemical components and anti-tumor activity of *Pleurotus eryngii* fruiting bodies increased with development to the mature stages [24].

Although the impacts of mushroom polysaccharides—in terms of molecular weight, polysaccharide composition, and structure—on gut microbiota modulation have been established in a review by Zhao et al. [25], the effects of the different stages and the link to the chemical compositions and consumption forms of mushroom have not yet been elucidated. Therefore, the current study aimed (1) to determine the chemical composition of the *P. atrovolvatus* fruiting body in both the egg and mature stages, which are commercially cultivated and consumed; and (2) to investigate gut microbiota-modulating activity, comparing both maturity stages (egg and mature fruiting body) and mushroom consumption forms (cooked mushroom and aqueous extract) to expand the current knowledge and promote the consumption of this unique and edible mushroom.

## 2. Materials and Methods

### 2.1. Raw Material

*P. atrovolvatus* was cultivated and provided by the Biotechnology Research and Development Office, the Department of Agriculture, the Ministry of Agriculture and Cooperatives, Bangkok, Thailand. The *P. atrovolvatus* samples at two stages (egg and mature fruiting body) were washed with water, dried in a hot-air oven (Memmert UN55; Büchenbach, Germany) at 60 °C for 18 h, and then ground (Blender, Tefal BL42S1; Rumilly, France) into powder. The particle size was approximately 150 μm.

### 2.2. Cooked Mushroom Preparation

Fresh *P. atrovolvatus* samples from both stages were washed with water, cut into small pieces, and boiled in hot water for 10 min. The cooked mushrooms from the egg (CME) and the mature fruiting body (CMF) were dried, ground into powder, and kept at −20 °C.

### 2.3. Hot Water Extraction

The mushroom aqueous extract was obtained using the method outlined in previous studies, with slight modifications [19,26]. The dried mushroom powder was mixed with deionized water at a ratio of 1:20 (*w*/*w*) and heated at 95 °C with magnetic stirring for 5 h. The mushroom solution was cooled to 37 °C, and α-amylase (30 U/mL, from porcine pancreatic type VI-B, A3176, Sigma; St. Louis, MO, USA) was added and incubated for another 1 h. Then, the mushroom solution was filtered, and the aqueous phase was mixed with 95% ethanol at a ratio of 1:4 (*v*/*v*) and kept at 4 °C overnight. Next, the mixture was centrifuged at 7000× *g* for 15 min, and the gel-like pellet was collected. The spent mushroom powder was used for a total of triple-repeated extraction. The resulting pellet was dried and ground into powder. The obtained mushroom aqueous extracts from the egg (MEE) and mature fruiting body (MEF) were kept at −20 °C.

### 2.4. Determination of Chemical Composition

#### 2.4.1. Proximate Analysis

A proximate analysis of the raw materials was determined using the methods outlined by AOAC 1995 [27]. The total protein content was determined using the Kjeldahl method. The total fat content was measured using Soxtec^TM^ (FOSS; Hilleroed, Denmark) with a petroleum ether solvent. The total ash content was determined by placing 1 g of sample in a crucible inside a furnace at 550 °C for 6 h. The moisture content was measured by calculating the weight loss after drying compared to the initial weight. The total carbohydrate content was calculated using the difference method.

#### 2.4.2. Total Dietary Fiber and Glucan Content

The total dietary fiber content of the raw materials was quantified according to an in-house method (TE-CH-076) based on AOAC (2019) 985.29 by the accredited Central Laboratory (Thailand) Co., Ltd. (Bangkok, Thailand). The amounts of total glucan and α- and β-glucan were quantified using a β-Glucan Assay Kit (Yeast & Mushroom, K-YBGL; Megazyme; Bray, Ireland) according to the manufacturer’s protocol. The β-glucan content was calculated by subtracting the α-glucan content from the total glucan content.

### 2.5. In Vitro Human Gut Fermentation

#### 2.5.1. Fecal Slurry Preparation

Fresh feces were collected from five healthy adult donors after obtaining their written consent. The healthy volunteers were 20–30 years old, with a body mass index ranging from 18.5 to 24.9 kg/m^2^ and no history of gastrointestinal disease. They had not received antibiotics in the past 3 months or probiotics/prebiotics in the previous 2 weeks before sample collection. The feces were collected in the morning on the same day. The fecal slurry was aseptically prepared under sterile conditions. The same amount of fecal sample from each donor was pooled in a stomacher bag (Stomacher^®^ 400 Classic Strainer Bag; Seward; West Sussex, UK). Then, the mixed fecal sample was diluted in sterile 0.1 M phosphate-buffered saline with a 30% (*v*/*v*) glycerol solution to obtain a 10% (*w*/*w*) fecal slurry, which was subsequently homogenized using a high-speed stomacher (Seward Stomacher 400 Circulator; West Sussex, UK) at 300 rpm for 3 min. The fecal slurry was split into aliquots, kept in a foil zip-lock bag at −20 °C, and used as the inoculum for three independent replications [28].

#### 2.5.2. In Vitro Human Fecal Batch Fermentation

A batch fermentation system was conducted in a water-jacket glass vessel maintained at 37 °C using a water-circulating system. The fecal fermentation method and composition of the fermentation medium were obtained from a previous study [29]. Each fermentation vessel contained 72 mL of sterile basal culture medium, which was pre-reduced overnight before fermentation. The pH was automatically controlled at 6.8 ± 0.1 by adding 0.1 M HCl or NaOH using a pH controller (Ferma 260; Electrolab; Gloucestershire, UK). Each vessel was stirred at 100 rpm. Sterile oxygen-free nitrogen gas was constantly supplied in the vessel to maintain an anaerobic condition during fermentation. Then, 0.8 g of mushroom samples (CME, CMF, MEE, or MEF) was added to the separate vessels to reach a final concentration of 1% (*w*/*v*). Inulin (INL) at a final concentration of 1% (*w*/*v*) was used as a positive control. No carbon source addition was performed as a blank control (CON). The substrates were incubated with a basal medium for 1 h before fermentation. Then, 8 mL of fecal slurry was added as an inoculum to reach 1% (*w*/*v*) of the fecal sample in each fermentation. The anaerobic fermentation was conducted for 24 h, and samples were collected.

#### 2.5.3. SCFAs, Phenol, and P-Cresol Determination

A fermented sample (1 mL) was mixed with 3 mL of internal standard solution (heptanoic acid at 5.04 μmol/mL of fermentation sample) and centrifuged at 2000× *g* at 4 °C for 10 min. Then, 300 μL of supernatant was mixed with 10 μL of 1 M phosphoric acid and passed through a 0.45 μm nylon filter into a 2 mL GC vial. The samples were stored at −20 °C until analysis. The SCFA content was analyzed using gas chromatography (TRACE 1310; Thermo Scientific; Waltham, MA, USA) equipped with a flame ionization detector and a DB-FFAP capillary column (30 m × 0.53 mm × 0.5 μm). The injector and detector temperatures were 210 °C, and the injection volume was 0.2 μL. The initial column temperature was held at 90 °C for 1 min, then heated at a rate of 20 °C/min to 190 °C and maintained for 2.5 min. The carrier gas was helium at a flow rate of 7.7 mL/min. A standard SCFA mixture was used for calculation, containing acetic, propionic, butyric, *i*-butyric, valeric, *i*-valeric, and hexanoic acids, together with standard phenol and *p*-cresol. All peak areas and other data were processed using Chromeleon software Version 7.2.10 (Sunnyvale, CA, USA). SCFAs, phenol, and *p*-cresol were identified and quantified based on the retention time and standard curve of standard compounds, respectively [30].

#### 2.5.4. Gut Microbiota Analysis

The DNA from the fermentation samples was extracted using a DNeasy PowerSoil Pro DNA Kit according to the manufacturer’s protocol (Qiagen; Germantown, MD, USA). The DNA samples were amplified using the 341F and 805R primers, targeting the V3-V4 variable regions with 2X sparQ HiFi PCR Master Mix (QuantaBio; Beverly, MA, USA). The primer sequences for the V3-V4 regions of the 16 s rRNA gene were F 5′-TCGTCGGCAGCGTCAGATGTGTATAAGCAGCCTACGGGNGGCWGCAG-3′ and R 5′ GTCTCGTGGGCTCGGAGATGTGTATAAGAGACAGGACTACHVGGTATCTAATCC-3′, respectively. The amplification conditions were an initial denaturation step at 98 °C for 2 min, followed by 30 cycles of denaturation at 98 °C for 20 s, and a single final extension step at 72 °C for 1 min. Subsequently, the 16S amplicons were purified using sparQ Puremag Beads (QuantaBio; Beverly, MA, USA) and indexed using 5 μL of each Nextera XT index primer in a 50 μL PCR reaction, followed by 10 cycles of the previous PCR condition. The final PCR products were cleaned, pooled, and diluted to a final loading concentration of 4 pM. Cluster generation and 250 bp paired-end read sequencing were performed on an Illumina MiSeq at the Omics Sciences and Bioinformatics Center (Chulalongkorn University; Bangkok, Thailand).

### 2.6. Statistical Analysis

All experiments were performed in three independent replications, with the results expressed as mean ± standard deviation values. The independent Student’s *t*-test and one-way analysis of variance (ANOVA) were applied to perform statistical difference analysis using the IBM Statistical Package for Social Sciences (SPSS) software version 26 (SPSS Inc.; Chicago, IL, USA). Duncan’s post hoc test was used to compare the means among treatments. A *p*-value < 0.05 was considered statistically significant.

The sequencing analysis was performed using R statistical software, version 4.3.1 (16 June 2023 ucrt), on the following platform: x86_64-w64-mingw32/x64 (64-bit) [31]. The relative abundance datasets with the lowest and highest values were normalized as zero and 100%, respectively. The results are shown as the mean ± standard error values of the mean. The Shapiro–Wilk test was performed to test the normality distribution of the normalized data. A mean comparison was performed using ANOVA and Tukey analyses for normally distributed data, while Kruskal–Wallis and Dunn’s tests were used for non-normal distributions. Non-parametric hypothesis testing used the Kruskal–Wallis post hoc Dunn test with R Package [32] and Fisheries Stock Assessment (FSA) [33]. Bonferroni correction was applied.

## 3. Results

### 3.1. Chemical Composition of P. atrovolvatus in Egg and Mature Stages

The dried fruiting bodies of *P. atrovolvatus* in the egg and mature stages were analyzed to compare their chemical components. The proximate constituents, total dietary fiber, and glucan contents are shown in Table 1. The total protein content of the fruiting bodies in the egg stage was 26.96%, which was significantly lower (*p* < 0.05) than in the mature stage at 29.80%. The ash and fat contents were approximately 8 and 0.4%, respectively. The total carbohydrate contents in the egg and mature stages were 52.01 and 49.53%, respectively, with the contents in the egg stage being significantly higher (*p* < 0.05). The total dietary fiber content in both stages was approximately 47%. The egg stage contained total glucan and β-glucan at 42.59% and 35.05%, which were significantly higher (*p* < 0.05) than in the mature stage at 32.12 and 22.92%, respectively. The α-glucan contents in the egg and mature stages were 7.52 and 9.20%, respectively, without a significant difference (*p* ≥ 0.05).

### 3.2. Cooked Mushroom and Mushroom Aqueous Extract

To compare mushroom consumption forms, cooked mushrooms and the mushroom aqueous extracts from both stages were analyzed in terms of their total protein and glucan contents, as shown in Table 2. The total protein values of MEE (23.99%) and MEF (31.32%) were significantly higher (*p* < 0.05) than for CME (22.86%) and CMF (21.30%), with MEF containing the highest protein content. The highest total glucan content was in CME (39.87%), followed by CMF (32.49%), MEF (29.51%), and MEE (22.33%), with β-glucan comprising the majority. CME contained the highest amount of β-glucan (33.28%), followed by MEF (26.77%), CMF (23.89%), and MEE (14.65%). The α-glucan of CME, CMF, and MEE ranged from 6 to 8%, with the lowest content observed in MEF (2.74%). The extraction yields of MEE and MEF were 15.38 and 12.20% (dry matter), respectively.

### 3.3. In Vitro Gut Fermentation

In vitro human fecal batch fermentation was performed to mimic colonic fermentation and to evaluate the gut microbiota-modulating activities of *P. atrovolvatus* cooked mushroom and its aqueous extract in both maturity stages. In this study, fermentations with mushroom samples (CME, CMF, MEE, or MEF) were compared with fermentations with INL (a commercial prebiotic) and CON (no added substrate).

#### 3.3.1. SCFA Production

The concentrations of SCFAs, as well as phenol and *p*-cresol, were determined at 24 h of fermentation, as shown in Table 3. The total SCFA contents promoted by CME (45.03 mM) and MEF (34.59 mM) were significantly higher (*p* < 0.05) than CON (26.47 mM) as the blank control. CME produced the highest level of total SCFAs among the other mushroom supplementations. The acetic acid concentrations of CME (32.04 mM), CMF (27.10 mM), and MEF (30.12 mM) were similar to that for INL (34.40 mM) (*p* ≥ 0.05) and significantly higher (*p* < 0.05) than for MEE (18.73 mM) and CON (19.52 mM). CME (5.17 mM) showed increased production of propionic acid to an equivalent level to INL (5.92 mM) (*p* ≥ 0.05). The levels of butyric acid production from fermentations with CME (6.76 mM) and MEE (6.84 mM) were significantly higher (*p* < 0.05) than with CON (2.93 mM), CMF (0.76 mM), and MEF (2.02 mM). Low concentrations of *i*-butyric acid were found in CME and MEE but not in CMF and MEF, while all samples produced low levels of valeric, *i*-valeric, and hexanoic acids. The phenol production was low in fermentation with MEE and MEF, while *p*-cresol was not found in any samples.

#### 3.3.2. Gut Microbiota Community

**Figure 1:**
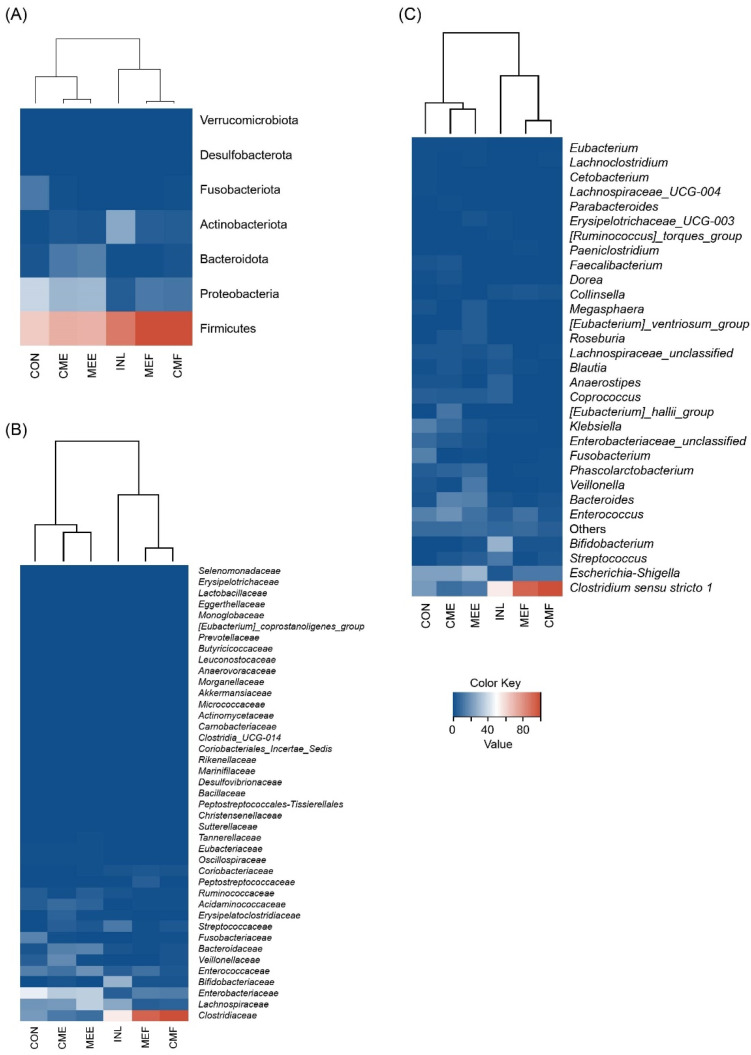
Heatmaps displaying the normalized relative abundances of the gut microbiota composition at the (**A**) phylum, (**B**) family, and (**C**) genus levels at 24 h of fermentation in the blank control (CON), inulin (INL), cooked mushroom from the egg stage (CME), cooked mushroom from the mature fruiting body stage (CMF), mushroom aqueous extract from the egg stage (MEE), and mushroom aqueous extract from the mature fruiting body stage (MEF). In each dataset, the lowest and highest values are normalized as 0 and 100%, respectively. At the genus level, only taxa with relative abundance levels higher than 1% are included.

The gut microbiota composition of an in vitro human fecal batch fermentation at 24 h was investigated using 16 s rRNA sequencing. Heatmaps showing the taxonomic abundance at the phylum family and genus levels are presented in Figure 1, with the color shading from red to blue indicating the highest to lowest relative abundance.

Figure 1A shows the phylum-level taxonomic composition. Significant increases (*p* < 0.05) in the relative abundance of Firmicutes and Actinobacteriota, commonly beneficial gut microbiota groups, were observed in the MEF- and CMF-supplemented fermentations compared to CON. In contrast, decreases in the relative abundance of Proteobacteria, Fusobacteriota, and Desulfobacterota, which are generally pathogenic bacterial groups, were also observed in the mushroom-supplemented fermentations. Relative to CON, there was a significant decrease (*p* < 0.05) in Proteobacteria in fermentations supplemented with MEF, CMF, and INL. The fermentations supplemented with INL and MEE showed significant decreases (*p* < 0.05) in Fusobacteria and Desulfobacterota. There were no significant differences (*p ≥* 0.05) in the relative abundance of Bacteroidota and Verrocomicrobiota among the tested samples compared to CON.

The family-level taxonomic composition is shown in Figure 1B. Compared to CON, fermentations supplemented with MEF and CMF showed significantly increased (*p* < 0.05) levels of relative abundance of *Bifidobacteriaceae* and *Enterococcaceae*. A significant increase (*p* < 0.05) in the relative abundance of *Streptococcaceae* was only observed with CME supplementation. These increasing changes in the relative abundance levels of all the mushroom-supplemented groups were similar to INL supplementation, with significantly increased (*p* < 0.05) relative abundances of the aforementioned bacterial families compared to CON. Moreover, changes in the relative abundance levels of bacterial families related to gut dysbiosis were observed. Compared to CON, the MEF- and CMF-supplemented groups showed significantly increased levels (*p* < 0.05) of the relative abundance of *Enterobacteriaceae* and *Veillonellaceae*. A significant reduction (*p* < 0.05) in *Fusobacteriaceae* was found with MEE supplementation. The INL-supplemented fermentation also followed the same trend as the mushroom supplementations. However, no significant differences (*p ≥* 0.05) were observed in the relative abundance levels of *Clostridiaceae* and *Lachnospiraceae* in any of the mushroom-treated groups, with these two bacterial families being the most abundant.

**Figure 2:**
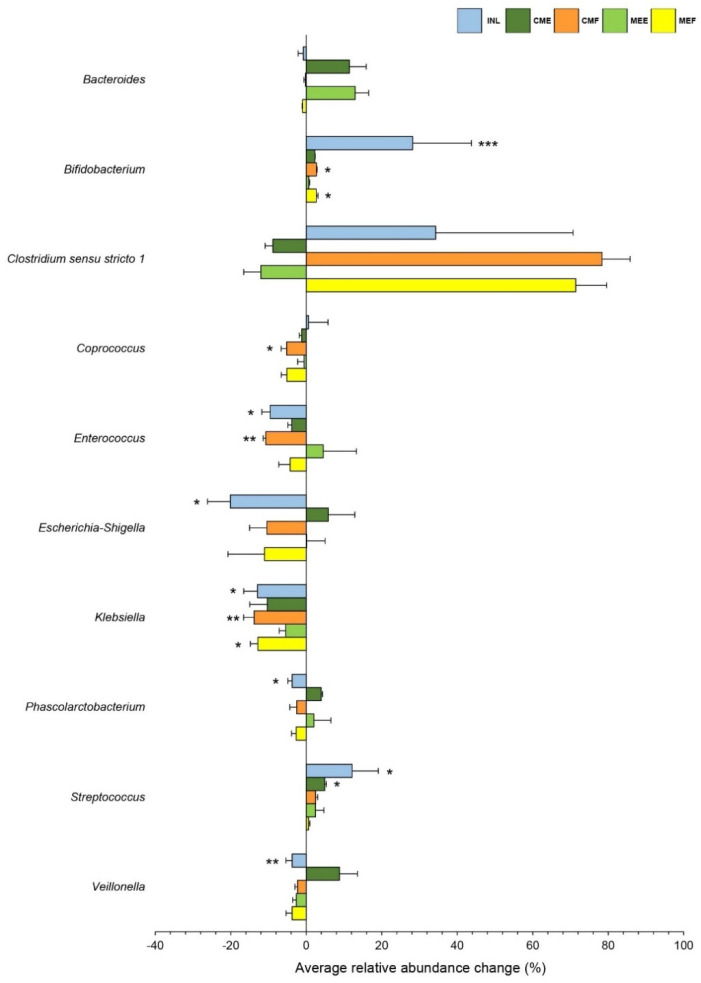
Genus-level changes in gut microbiota at 24 h of fermentation supplemented with inulin (INL), cooked mushroom from the egg stage (CME), cooked mushroom from the mature fruiting body stage (CMF), mushroom aqueous extract from the egg stage (MEE), and mushroom aqueous extract from the mature fruiting body stage (MEF) compared to the blank control (CON). The results shown are the mean ± standard error values of the mean. Only the top-ten genera with an abundance greater than 1% are shown (* *p* < 0.05, ** *p* < 0.01, *** *p* < 0.001, compared to CON).

The effects of mushroom-supplemented fermentations on the gut microbiota population were also observed at the genus level (Figure 1C). Changes in the relative abundance levels of the top-ten genera with an abundance of more than 1% (*Clostridium sensu stricto 1*, *Escherichia-Shigella*, *Enterococcus*, *Bacteroides*, *Bifidobacterium*, *Klebsiella*, *Streptococcus*, *Phascolarctobacterium*, *Coprococcus*, and *Veillonella*) are shown in Figure 2. Compared to CON, all mushroom-supplemented fermentations promoted the growth of *Bifidobacterium*, *Streptococcus*, and *Clostridium sensu stricto 1*, except for CME and MEE, where the relative abundances of *Clostridium sensu stricto 1* showed a decreasing trend. The INL-supplemented fermentation significantly increased (*p* < 0.05) the growth of *Bifidobacterium* and *Streptococcus* probiotic bacteria but not *Clostridium sensu stricto 1*. No significant changes (*p ≥* 0.05) in *Bacteroides* were observed in CMF, MEF, and INL; however, increases were found in CME and MEE compared to the CON group. Only CMF-supplemented fermentation significantly reduced (*p* < 0.05) the growth of the *Coprococcus* population. Compared to CON, all mushroom-treated conditions did not show significant differences in the relative abundance of the *Phascolarctobacterium* population, but a significant decrease (*p* < 0.05) was exclusively found in INL. A significant reduction (*p* < 0.05) in the relative abundance of the *Enterococcus* was observed in CMF- and INL-supplemented fermentations but not in the others compared to CON.

Apart from potentially beneficial bacteria, changes in pathogenic bacteria were also observed in this study. Relative to CON, all mushroom supplementations reduced the relative abundance of *Escherichia-Shigella*, *Klebsiella*, and *Veillonella*, with CMF and MEF significantly inhibiting (*p* < 0.05) the growth of *Klebsiella*. Similarly, significant reductions (*p* < 0.05) in these pathogenic genera were found in the INL-supplemented fermentation. However, non-significant increases (*p ≥* 0.05) of *Escherichia-Shigella* and *Veillonella* were observed in the CME-supplemented fermentation.

## 4. Discussion

The present study illustrated the chemical composition of dried *P. atrovolvatus* fruiting bodies. The main components of this mushroom were carbohydrates and protein, followed by ash and fat. The protein content of *P. atrovolvatus* was comparable with other edible wild-grown mushrooms, which have a reported range of 12–29% [34]. The results indicated that almost all of the carbohydrate content of this mushroom was dietary fiber. The total glucan, α-glucan, and β-glucan contents of this mushroom in both stages were in the same range as other commercially cultivated and wild mushrooms, at 23–83%, 1–16%, and 18–73%, respectively [35]. The β-glucan/total glucan ratios of this mushroom in the egg and mature stages were 82.34 and 71.33%, respectively, indicating that β-glucan made up the main glucan component in this mushroom. In addition, the β-glucan contents in the egg and mature stages were 35.07 and 22.92%, accounting for 73.67 and 48.96% of the total dietary fiber, respectively. Hence, β-glucan was the major dietary fiber component of the *P. atrovolvatus* fruiting body. Additionally, the higher β-glucan content in the egg stage might be due to the different expression levels of glucan synthesis/hydrolysis-associated genes during fruiting body development. Another study demonstrated that the expression of glucan synthesis-associated enzyme genes in the egg stage was higher than in the mature stage because it was expressed when preparing the glucan precursor and synthesizing the glucan chains. The expression level of glucanase in the mature stage to degrade glucan in the cell wall was higher than in the egg stage to support the elongation and opening of the fruiting body [36].

Generally, mushrooms are consumed as cooked mushrooms and mushroom extracts. Hot water has been effectively used to extract mushroom polysaccharides, including β-glucan, which can modulate the gut microbiota and exert prebiotic properties [37,38]. Therefore, to imitate dietary consumption, *P. atrovolvatus* mushrooms from both maturity stages were cooked in hot water and extracted using hot water. According to the results, the total protein contents of MEE and MEF were significantly higher than those of CME and CMF. In the mushroom cell wall, protein is combined with polysaccharides as a complex structure called glycoprotein [39]. Hot water extraction was performed to break the cell wall structure, and the protein–polysaccharide complex was then isolated and precipitated as an aqueous extract [40]. Hence, the high protein content in the mushroom aqueous extracts might have been glycoprotein from the cell wall. Additionally, the total protein contents of CME and CMF were lower than the raw materials at 26.96% for the egg and 29.80% for the mature stage. Our results are consistent with other studies, which reported a reduced crude protein content through boiling due to protein denaturation, solubilization, and the leaching out of nitrogenous substances [41].

In this study, α-amylase was used to remove starch by cleaving α-1,4 glycosidic bonds in the α-glucan backbone during extraction [42], resulting in reduced α-glucan content and an increased concentration of β-glucan in the mushroom aqueous extract. Compared to raw materials, a decrease in α-glucan was observed in MEF but not in MEE. This indicated that hot water extraction, coupled with α-amylase, was more effective in isolating β-glucan from the raw material of the mature stage than from the egg stage due to the firm and compact structure of the egg stage [36], while traditional hot water extraction might not have enough efficacy to break the cell wall. However, the extraction yields of this study were higher than in another study that reported a yield of crude polysaccharide from *P. atrovolvatus* of approximately 6% [19], possibly due to our triple-repeated extraction, which improved the yield. In addition, Shao et al. reported a higher extraction yield of crude polysaccharides from the egg stage than from the mature stage, at 8.12 and 3.72%, respectively [36].

SCFAs are the main gut-microbe-derived metabolites contributing to human health benefits, especially gut immune-modulating activities [43]. In this study, the main SCFAs produced in all mushroom fermentations were acetic acid, propionic acid, and butyric acid, with acetic acid being the major component. The fermentation supplemented with INL as a positive control had the highest levels of total SCFAs, acetic acid, propionic acid, and butyric acid, as inulin is a soluble dietary fiber and is regarded as a prebiotic, which can be utilized by the gut microbiota [44]. Phenol and *p*-cresol are toxic bacterial metabolites, mainly observed from protein fermentation. These compounds are produced through microbial metabolism of the aromatic amino acid tyrosine in the colon [45], which is associated with bowel diseases and the consumption of meat protein [46]. Although the current samples contained 20–30% protein, low production of phenol was only found in MEE and MEF, and there was no observation of *p*-cresol in any samples. This was possibly due to the low tyrosine content in mushrooms, reported in the range of 1–10% in wild edible mushrooms [47], while meat and meat products were found to represent an average of 38% [48].

The β-glucan content present in each sample may influence the production of SCFAs. The gut microbiota converts β-glucan to glucose molecules via the activation of carbohydrate-active enzymes, such as β-glucanase, glycoside hydrolases, and polysaccharide lyases, which subsequently serve as substrates for SCFA synthesis through microbial anaerobic fermentation [49]. Thus, the SCFAs produced in this study likely resulted from these mechanisms, as total SCFAs levels were promoted and correlated with the β-glucan content in all of the mushroom samples. Our current findings concurred with other studies, demonstrating that fermentation supplemented with β-glucan and polysaccharides from mushrooms promoted the production of SCFAs, of which acetic acid, propionic acid, and butyric acid were the most abundant in an in vitro human fecal batch fermentation system. In the current study, the level of total SCFAs with the mushroom treatments (29–45 mM) was slightly higher than in fermentations supplemented with β-glucan extracts from *Schizophylum commune* Fr and *Auricularia auricula* mushrooms, which were in the range of 27–39 mM. Our results showed higher acetic acid concentrations (18–32 mM) than the other studies (8–23 mM). Similarly, the production of propionic and butyric acids (1–6 mM) in our study concurred with other studies (1–10 mM) [50,51,52,53].

Regarding gut microbiome modulation, fermentations with mushroom samples increased the relative abundance of probiotic bacteria, including *Bifidobacterium*, *Streptococcus*, and *Clostridium sensu stricto 1*, showing the same trend as INL. The increased growth of probiotic groups in the mushroom-supplemented fermentations might result from the presence of β-glucan in the samples, as these three probiotic genera could utilize β-glucan as a carbon source, leading to increased acetic acid production [54,55,56]. These results correlated with higher acetic acid levels than CON. The increased levels of the relative abundance of *Bacteroides*, a primary glycan degrader [57], were only observed in samples from the egg stages (CME and MEE). The mushroom-supplemented fermentation could not promote the growth of *Phascolarctobacterium* and *Coprococcus*, which are SCFA-producers [58,59]. In addition, the INL- and CMF-supplemented fermentations inhibited the growth of the *Enterococcus*, which is mainly known as a commensal lactic acid bacterium and has probiotic potential; however, it remains controversial as an emerging pathogenic bacteria involved in antibiotic resistance and bowel diseases [60,61]. Overall, our findings concurred with other studies, demonstrating that mushrooms and/or polysaccharides from *Pleurotus eryngii*, *P. ostreatus*, *Hericium erinaceus*, and *Wolfiporia cocos* increased the abundance of beneficial and SCFA-producing bacteria in an in vitro gut fermentation system [62,63,64]. Increases in the relative abundance levels of probiotics and SCFA-producing bacterial groups were also correlated with SCFA production, showing that mushroom-supplemented fermentations promoted SCFA production compared to the blank control.

In addition to beneficial bacteria, the mushroom-supplemented groups inhibited the relative abundance of *Escherichia-Shigella*, *Klebsiella*, and *Veillonella*, which are potentially involved in gut dysbiosis and gastrointestinal tract diseases [65,66,67]. Other studies have reported that the numbers of *Escherichia-Shigella* and *Veillonella* in patients with Crohn’s disease were higher than in healthy groups and were strongly correlated with a reduction in SCFA-producing bacteria [68]. Our results concurred with other studies indicating that mushroom polysaccharides reduced the relative abundance levels of these three harmful bacteria [63,64,69].

Interestingly, the heatmaps combined with cluster analysis indicated that the relative abundance changes in MEF and CMF were similar to that of INL. At the same time, CME and MEE correlated with CON at all taxonomic levels. Our findings showed that gut microbiota modulation of the cooked mushroom and the aqueous extract from the mature stage were more similar to inulin than to the egg stage, possibly due to the different structural complexity in each mushroom maturity stage [36]. Since differences in polysaccharide structure might affect the utilization of gut microbiota, further studies should compare glycosidic linkages and configurations, molecular weight, and monosaccharide compositions across maturity stages.

## 5. Conclusions

*P. atrovolvatus*, both as a cooked mushroom and in aqueous extracts, showed promising gut health benefits by promoting the production of short-chain fatty acids, particularly acetic acid, propionic acid, and butyric acid. Additionally, they enhanced the growth of beneficial bacteria in the genera *Bacteroides*, *Bifidobacterium*, *Clostridium sensu stricto 1*, and *Streptococcus*, while inhibiting potentially pathogenic bacteria, such as *Escherichia-Shigella*, *Klebsiella*, and *Veillonella*. Compared to the egg stage, both the cooked mushroom and the aqueous extract from the mature stage exhibited superior prebiotic potential to modulate gut microbiota. Further characterization of this mushroom’s polysaccharide structure should consider its different maturity stages. Based on our in vitro findings, future studies on gut health benefits should include in vivo and clinical studies to determine the effective dosages, assess toxicology, and understand the long-term impacts.

## Figures and Tables

**Table 1:** The chemical components of dried *P. atrovolvatus* in egg and mature stages (% dry matter).

Constituent	Egg	Mature Fruiting Body
Moisture	12.47 ± 0.20	12.59 ± 0.16
Protein	26.96 ± 0.03	29.80 ± 0.02 *
Ash	8.09 ± 0.02 *	7.66 ± 0.05
Fat	0.47 ± 0.00 *	0.42 ± 0.02
Carbohydrate	52.01 ± 0.21 *	49.53 ± 0.21
Total dietary fiber	47.61 ± 0.03 *	46.79 ± 0.11
Total glucan	42.59 ± 0.35 *	32.12 ± 1.47
α-Glucan	7.52 ± 1.23	9.20 ± 0.91
β-Glucan	35.07 ± 1.40 *	22.92 ± 0.81

Values are expressed as means ± SD. * indicate a significant (*p* < 0.05) difference between two stages in the same row. Two independent replications were performed for all analyses except for the glucan contents, which were analyzed in triplicate.

**Table 2:** The total protein and glucan contents of cooked mushrooms and the mushroom aqueous extract from egg and mature stages (% dry matter).

Constituent	CME	CMF	MEE	MEF
Total protein	22.86 ± 0.09 ^c^	21.30 ± 0.09 ^d^	23.99 ± 0.03 ^b^	31.32 ± 0.05 ^a^
Total glucan	39.87 ± 0.28 ^a^	32.49 ± 0.87 ^b^	22.33 ± 0.27 ^d^	29.51 ± 0.27 ^c^
α-glucan	6.59 ± 0.36 ^c^	8.60 ± 0.72 ^a^	7.68 ± 0.18 ^b^	2.74 ± 0.02 ^d^
β-glucan	33.28 ± 0.38 ^a^	23.89 ± 0.22 ^c^	14.65 ± 0.09 ^d^	26.77 ± 0.29 ^b^

Abbreviations: CME, cooked mushroom from the egg stage; CMF, cooked mushroom from the mature fruiting body stage; MEE, mushroom aqueous extract from the egg stage; MEF, mushroom aqueous extract from the mature fruiting body stage. Values (means ± SD) within each row with different lowercase superscripts are significantly (*p* < 0.05) different.

**Table 3:** SCFA concentrations (mM) at 24 h of in vitro human gut fermentation.

SCFA	CON	INL	CME	CMF	MEE	MEF
Total	26.47 ± 1.52 ^d^	55.13 ± 7.48 ^a^	45.03 ± 3.60 ^b^	30.24 ± 0.63 ^cd^	29.58 ± 3.15 ^cd^	34.59 ± 2.73 ^c^
Acetic	19.52 ± 1.14 ^b^	34.40 ± 7.25 ^a^	32.04 ± 0.08 ^a^	27.10 ± 1.86 ^a^	18.73 ± 5.54 ^b^	30.12 ± 2.11 ^a^
Propionic	2.00 ± 0.35 ^b^	5.92 ± 2.47 ^a^	5.17 ± 1.56 ^a^	1.83 ± 1.07 ^b^	2.57 ± 0.80 ^b^	1.93 ± 0.25 ^b^
Butyric	2.93 ± 0.03 ^c^	13.82 ± 0.66 ^a^	6.76 ± 1.81 ^b^	0.76 ± 0.11 ^d^	6.84 ± 1.71 ^b^	2.02 ± 0.52 ^cd^
*i*-butyric	0.06 ± 0.00 ^b^	0.07 ± 0.00 ^c^	0.11 ± 0.03 ^b^	ND	0.20 ± 0.01 ^a^	ND
Valeric	0.09 ± 0.01 ^c^	0.19 ± 0.04 ^b^	0.10 ± 0.04 ^c^	0.04 ± 0.01 ^d^	0.42 ± 0.00 ^a^	0.02 ± 0.01 ^d^
*i*-valeric	0.06 ± 0.00 ^bc^	0.11 ± 0.06 ^ab^	0.08 ± 0.03 ^abc^	0.05 ± 0.01 ^c^	0.14 ± 0.01 ^a^	0.07 ± 0.03 ^bc^
Hexanoic	ND	0.62 ± 0.26 ^ab^	0.76 ± 0.09 ^a^	0.46 ± 0.22 ^ab^	0.68 ± 0.17 ^ab^	0.43 ± 0.04 ^b^
Phenol	ND	0.19 ± 0.07 ^b^	ND	ND	0.18 ± 0.04 ^b^	0.28 ± 0.03 ^a^
*p*-cresol	ND	ND	ND	ND	ND	ND

Abbreviations: CON, blank control; INL, inulin; CME, cooked mushroom from the egg stage; CMF, cooked mushroom from the mature fruiting body stage; MEE, mushroom aqueous extract from the egg stage; MEF, mushroom aqueous extract from the mature fruiting body stage. Values (mean ± SD) within each row with different lowercase superscripts are significantly (*p* < 0.05) different. ND = not detected.

## Data Availability

The dataset generated in this research is available from the corresponding author upon reasonable request. The data are not publicly available due to privacy restrictions.
